# Association of Discharge to Home vs Institutional Postacute Care With Outcomes After Lower Extremity Joint Replacement

**DOI:** 10.1001/jamanetworkopen.2020.22382

**Published:** 2020-10-23

**Authors:** Robert E. Burke, Anne Canamucio, Elina Medvedeva, Eric L. Hume, Amol S. Navathe

**Affiliations:** 1 Center for Health Equity Research and Promotion, Corporal Michael Crescenz VA Medical Center, Philadelphia, Pennsylvania; 2 Division of General Internal Medicine, Department of Medicine, University of Pennsylvania Perelman School of Medicine, Philadelphia; 3 Department of Orthopaedic Surgery, University of Pennsylvania, Penn Musculoskeletal Center, Philadelphia; 4 Department of Medical Ethics and Health Policy, University of Pennsylvania Perelman School of Medicine, Philadelphia

## Abstract

**Importance:**

Changes in financial incentives have led to more patients being discharged home than to institutional forms of postacute care, such as skilled nursing facilities (SNFs), after elective lower extremity total joint replacement (LEJR).

**Objective:**

To evaluate the association of this change with hospital readmissions, surgical complications, and mortality.

**Design, Setting, and Participants:**

This cohort study used cross-temporal propensity-matching to identify 104 828 adult patients who were discharged home following LEJR between 2016 and 2018 (after changes in financial incentives) and 84 121 adult patients discharged to institutional forms of postacute care (eg, SNFs) between 2011 and 2013 (before changes in financial incentives). A difference-in-differences design was used to compare differences in outcomes between these groups to a propensity-matched group of patients discharged to institutional postacute care in both periods. Data were collected from Pennsylvania all-payer claims database, which includes all surgical procedures and hospitalizations across payers and hospitals in Pennsylvania. Data were analyzed between August 2019 and February 2020.

**Exposures:**

Type of postacute care (home, including home with home health vs institutional postacute care, including SNF, inpatient rehabilitation facilities, and long-term acute care hospitals).

**Main Outcomes and Measures:**

Main outcomes were 30- and 90-day hospital readmissions, LEJR complication rates, and mortality rates.

**Results:**

Of 189 949 patients, 113 981 (60.0%) were women, and 83 444 (43.9%) were aged 40 to 64 years. The rate of discharge home increased from 63.6% (54 097 of 85 121) in 2011 to 2013 to 78.4% (82 199 of 104 828) in 2016 to 2018. In the adjusted difference-in-differences comparison, matched patients discharged home in 2016 to 2018 had significantly lower 30-day (difference, −2.9%; 95% CI, −4.2% to −1.6%) and 90-day (difference, −3.9%; 95% CI, −5.8% to −2.0%) readmission rates compared with similar patients sent to institutional postacute care in 2011 to 2013. Surgical complication and mortality rates were unchanged. Results were similar across payers and across hospital bundled payment participation status.

**Conclusions and Relevance:**

In this cohort study, increases in discharges home following LEJR surgery did not seem to be associated with increased harm during a period in which changes in financial incentives likely spurred observed changes in postacute care.

## Key Points

**Question:**

How have clinical outcomes changed for adults undergoing lower extremity total joint arthroplasty whose postdischarge care has shifted from institutional postacute care settings to home because of changes in financial incentives?

**Findings:**

This cohort study of 189 949 adult patients who underwent lower extremity joint replacement procedures in Pennsylvania used a matched difference-in-differences approach. Adults discharged home between 2016 and 2018 had fewer hospital readmissions than propensity-matched adults who had been discharged to institutional postacute care between 2011 and 2013, but no differences in surgical complications or mortality were observed.

**Meaning:**

In this study, there was no association with worsening outcomes for adults who were discharged home following joint arthroplasty and were clinically similar to patients discharged to institutional postacute care before financial incentives changed.

## Teaser

This cohort study evaluates the association of discharge home vs discharge to institutional postacute care with outcomes following elective lower extremity total joint replacement.

## Introduction

What level of postacute care support is provided to patients after elective lower extremity joint replacement (LEJR) is an increasingly important clinical and policy issue. More than 1 million LEJR operations are performed annually in the United States, and this volume is expected to increase as the population ages. The growth in the volume of LEJR and associated costs has led the Centers for Medicare & Medicaid Services (CMS) to reform payments for LEJR, which has changed the landscape of postacute care following surgery.^1^

The Bundled Payments for Care Improvement (BPCI) and Comprehensive Care for Joint Replacement (CJR) models have shown reductions in episode costs primarily through reduced use of expensive forms of postacute care (eg, skilled nursing facility [SNF] care) in favor of discharges home (with or without home health care).^2,3,4,5,6,7,8^ The Hospital Readmissions Reduction Program (HRRP) announced inclusion of elective hip and knee replacements as a targeted condition in 2013 and instituted penalties for 30-day readmissions starting in 2015.^9^ Early evaluations of these individual programs have not shown evidence of harm, even in frail LEJR populations, although long-term effects remain unstudied.^4,6,10,11,12,13^

Because BPCI started in late 2013, HRRP had important milestones for LEJR in 2013 and 2015, and CJR started in 2016, the period from 2013 to 2016 had dramatic changes in financial incentives for LEJR care. Although prior evaluations have identified the consequences of individual programs, it is likely that patient care patterns changed more broadly during this period, particularly for postacute care. In addition, these changes may extend to other payers, hospitals, and outpatient surgery centers performing LEJR who may not be participating in bundled payments, but these effects are not well described. Thus, the totality of how health reform efforts on LEJR affected postacute care patterns remains unknown.

To address this evidence gap, our study had 2 objectives: first, to describe the secular trends in postacute care use across all payers and hospitals in Pennsylvania, comparing the time period before these changes in financial incentives (2011-2013) with the period after these changes (2016-2018), and, second, to examine the association between changes in postacute care use and changes in hospital readmission, surgical complications, or mortality rates. We hypothesized that patients now being discharged home who would have been discharged to institutional postacute care before payment reform (ie, patients for whom the policies led clinicians to switch the site of postacute care to home) would be associated with worse outcomes.

## Methods

Because our data set was deidentified, our study was considered exempt by the University of Pennsylvania institutional review board, and informed consent was not required. This study followed Strengthening the Reporting of Observational Studies in Epidemiology (STROBE) reporting guideline.

### Data Source

We used Pennsylvania’s Health Care Cost Containment Council (PHC4) data, comparing July 1, 2011, to June 30, 2013 (ie, the preperiod), with July 1, 2016, to June 30, 2018 (ie, the postperiod). PHC4 captures all discharges from Pennsylvania hospitals, including specialty surgical hospitals, as well as all ambulatory surgical procedures, including LEJR, regardless of payer. We linked these data to mortality data from the Pennsylvania Department of Health vital statistics files. The preperiod preceded the start of LEJR-oriented payment reforms that began in 2013.^8^ The postperiod was the most recent period available in PHC4 data.

### Participants

Our sample included adults aged 18 years and older who underwent elective total hip or knee arthroplasty as an inpatient or outpatient in Pennsylvania (identified using diagnosis-related groups codes 469 and 470 for inpatients and *Common Procedural Terminology* codes 27447 and 27130 for outpatients). Patients undergoing arthroplasty for other reasons (ie, revision, trauma, in the setting of malignant neoplasms) were excluded. We included all hospitals and outpatient surgery centers that performed elective hip or knee arthroplasty in Pennsylvania during the period.

### Independent Variables

Discharge location was captured as home, home with home health, SNF care, inpatient rehabilitation facility (IRF) care, and long-term acute care hospital (LTACH) in PHC4 data. We dichotomized these variables into discharges home (home and home with home health) and discharges to institutional postacute care (including SNF, IRF, and LTACH). Patient characteristics included age, sex, race and ethnicity, comorbidities (captured as Charlson Comorbidity Index score using hospital admission and discharge diagnoses), primary payer, and index hospital length of stay. Race and ethnicity data were captured administratively by each hospital contributing data as part of routine clinical care; we report race and ethnicity information to establish the generalizability of results and minimize bias owing to unmeasured confounding. Hospital characteristics included number of beds, region (eFigure in the Supplement), and facility type (specialty acute care, such as orthopedic and psychiatric hospitals, general acute care, and ambulatory surgery centers).

### Outcomes

We evaluated hospital readmission rates (30-day and 90-day), mortality (30-day and 90-day), and LEJR postsurgical complication rates. The LEJR complication rate was defined using *International Classification of Diseases, Ninth Revision *(*ICD*-*9*) (2011-2013) and *International Statistical Classification of Diseases and Related Health Problems, Tenth Revision *(*ICD*-*10*) (2016-2018) codes, per the CJR rules;.^14^ These rules specify a complication is defined using *ICD*-*9* or *ICD*-*10* codes for acute conditions occurring during the index admission or within 7 days of hospital discharge, including acute myocardial infarction, death, mechanical complications (such as a broken prosthesis), joint or surgical wound infection, pneumonia, pulmonary embolism, sepsis, and surgical site bleeding. We included complications after hospital discharge as an outcome but adjusted for complications that occurred during the index stay.

### Analysis

#### Conceptual Approach

Our goal was to compare outcomes for adults who were discharged home in the postperiod (ie, patients for whom the policies led clinicians to switch the site of postacute care to home in the postperiod) with similar adults discharged to institutional postacute care in the preperiod (Figure). We used a difference-in-differences cross-temporal matching design to compare clinically similar patients (with respect to requirements for postacute care and its intensity, using propensity matching) and to account for secular trends (using a difference-in-differences approach). This design has been used previously to examine large secular changes in practice over time in response to financial incentives.^15^

**Figure.**
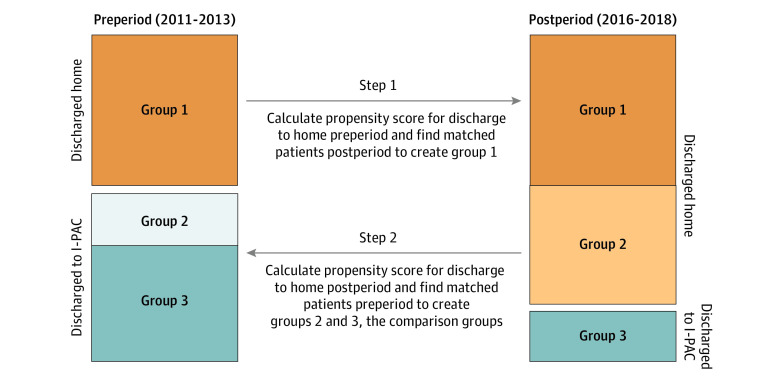
Cross-Temporal Matching Design Steps of the analytic design are presented. Group 1 is created by propensity matching patients discharged home in the preperiod with those discharged home postperiod. This group is not used further in the analysis because they never received institutional forms of postacute care (I-PAC; including care in skilled nursing facilities, inpatient rehabilitation facilities, and long-term acute care hospitals). Groups 2 and 3 are used in the difference-in-differences comparison and are created by propensity matching patients discharged home in the postperiod with those discharged to I-PAC in the preperiod (group 2) and patients discharged to I-PAC during both periods (group 3).

#### Propensity Matching

The first step in the design was to create propensity-matched groups for comparison in difference-in-differences analysis. We used propensity matching with replacement to minimize the differences between patients in the preperiod and postperiod. The propensity scores were calculated using multivariable logistic regression with patient demographic characteristics, comorbidities, index hospital length of stay, hospital size, region, and type of hospital as covariates. We first calculated the propensity to be discharged home in the preperiod cohort and applied this model to the patients in the postperiod to create matches of observably similar patients who were discharged home in both periods (ie, group 1) (Figure). These matched patients were excluded from further analysis because none used institutional postacute care in either period and were thus unlikely to have their postdischarge care associated with financial incentives (ie, patients among these matches in the postperiod were unlikely to be those switched to home from institutional postacute care). They were also likely to be clinically different from our population of interest.

We then calculated the propensity to be discharged home for each remaining patient in the postperiod and used this model’s coefficients, applied to each remaining patient in the preperiod, to generate the propensity to be discharged home (although we knew the setting to which the patient was actually discharged). We then used these scores to create our 2 remaining groups: a matched group of patients discharged home in the postperiod and to institutional postacute care in the preperiod (ie, group 2) (Figure) and a matched group of patients discharged to institutional postacute care in both periods (ie, group 3) (Figure). These are our comparison groups of interest. Of note, few patients underwent LEJR surgery in both time periods, and we did not match within patient (ie, the same patient undergoing 2 different surgeries in the 2 periods).

We tested the propensity score matching in 2 ways. First, we examined *C* statistics to assess the accuracy of the estimation of discharge destination in both periods. Second, we calculated the standardized mean differences between groups after matching to examine balance. The *C* statistic was greater than 0.8 for each propensity match, and standardized mean differences were less than 10 for all variables other than high Charlson Comorbidity Index score, suggesting that groups were similar on observed characteristics.

### Statistical Analysis

We first described the characteristics of patients discharged to home and institutional postacute care during both periods. We calculated how rates of discharge to different forms of postacute care changed over the 2 periods, including how this varied by payer and by hospital participation in bundled payments (BPCI or CJR). The difference-in-differences analysis used a multivariable logistic regression model (because all outcomes were binary) using a single specification for all outcomes. This model compared changes in outcomes between matched patients discharged home in the postperiod with those discharged to institutional postacute care in the preperiod (group 2) and with the pre-post changes for those discharged to institutional postacute care in both periods (group 3). We calculated our difference-in-differences analysis as (postperiod group 2 − preperiod group 2) − (preperiod group 3 − postperiod group 3), using robust standard errors to adjust for clustering of patients within hospitals. In essence, group 3 represents changes over time in outcomes owing to secular trends; changes in outcomes in group 2 are compared with these secular trends to see if there had been more change than expected.

We also conducted 4 sensitivity analyses. First, we analyzed whether findings were similar in a Medicare-only cohort, which included redoing the propensity matching and then conducting an additional difference-in-differences analysis. Second, we identified whether results differed by hospitals’ participation in bundled payments (BPCI or CJR). We identified their participation in BPCI (for the LEJR episode) or CJR on a quarterly basis in the postbundle period using the CMS BPCI analytic file, as in prior evaluations.^5,6^ Third, we identified how our findings varied by age and comorbidity burden of the propensity-matched overall cohort, comparing results in the oldest and youngest quartiles as well as the lowest and highest Charlson Comorbidity Index score quartiles. Fourth, we examined sensitivity of our analysis to a hypothetical unmeasured confounder to examine robustness of our results. This procedure calculated an E-value.^16^ The E-value is defined as the minimum strength of association an unmeasured confounder in our data would need to have with both the treatment and outcome to explain away our findings, conditional on the measured covariates. Small E-values mean little unmeasured confounding would need to be present to explain away an effect estimate.

Data analysis was conducted in SAS version 9.4 (SAS Institute). Statistical significance was set at *P* < .05, and all tests were 2-tailed.

## Results

Our overall cohort included 85 121 patients who underwent LEJR in the preperiod and 104 828 in the postperiod. Of these 189 949 patients, 113 981 (60.0%) were women, 171 442 (90.3%) were White individuals, and 83 444 (43.9%) were aged 40 to 64 years. Overall, 106 843 patients (56.2%) had Medicare, and 73 807 (38.9%) had commercial insurance. Overall, 188 259 operations (99.1%) in both periods took place in the inpatient setting at 166 hospitals across Pennsylvania; 46 hospitals (27.7%) participated in BPCI or CJR for at least 1 quarter. Most procedures took places in large, general hospitals (≥325 beds, 81 310 [42.8%]; general hospitals, 173 202 [91.2%]). There was an absolute increase of 14.8% (from 63.6% [54 097 of 85 121] to 78.4% [82 199 of 104 828]) in the number of patients discharged home in the postperiod compared with the preperiod, while discharges to institutional postacute care decreased 14.9% (from 36.5% [31 024 of 85 121] to 21.6% [22 692 of 104 828]). Patients discharged home in the postperiod left the hospital more than 2 full days earlier than in the preperiod (mean [SD] length of stay, 1.9 [1.2] days vs 4.1 [2.6] days), despite similar comorbidity scores (mean [SD] Charlson Comorbidity Index score, 0.6 [1.1] vs 0.5 [0.9]), while those discharged to institutional postacute care in the postperiod stayed a full day longer than those in the preperiod (mean [SD] length of stay, 3.9 [2.6] days vs 2.9 [1.3] days) (Table 1). The increase in discharges home was similar in hospitals that did and did not participate in BPCI and CJR and extended across every payer (Table 2). Use of home health remained similar across periods; the increase in home discharges was among patients discharged without home services.

**Table 1.** Sample Cohort Before Propensity Matching, Stratified by Postacute Care Location and Period

Characteristic	Patients, No. (%)						
	Preperiod, 2011-2013			Postperiod, 2016-2018			
	Discharged home (n = 54 097)	Discharged to IPAC (n = 31 024)	Total (n = 85 121)		Discharged home (n = 82 199)	Discharged to IPAC (n = 22 629)	Total (n = 104 828)
Age, y							
18-39	824 (1.5)	81 (0.3)	905 (1.1)		841 (1.0)	47 (0.2)	888 (0.9)
40-64	28 805 (53.3)	6780 (19.1)	35 585 (41.8)		37 999 (46.2)	3710 (16.4)	41 709 (39.8)
65-79	21 618 (40.0)	13 682 (44.1)	35 300 (41.5)		38 022 (46.3)	10 122 (44.7)	48 144 (45.9)
80-100	2850 (5.3)	10 481 (33.8)	13 331 (15.7)		5337 (6.5)	8750 (38.7)	14 087 (13.4)
Men	24 415 (45.1)	9093 (29.3)	33 508 (39.4)		35 844 (43.6)	6616 (29.2)	42 460 (40.5)
Race							
White	49 365 (91.3)	27 456 (88.5)	76 821 (90.3)		74 717 (90.9)	19 904 (88.0)	94 621 (90.3)
Black	2400 (4.4)	2588 (8.3)	4988 (5.9)		4633 (5.6)	2033 (9.0)	6666 (6.4)
Other	1113 (2.1)	545 (1.8)	1658 (2.0)		1200 (1.5)	337 (1.5)	1537 (1.5)
Unknown	1219 (2.3)	435 (1.4)	1654 (1.9)		1649 (2.0)	355 (1.6)	2004 (1.9)
Hispanic	347 (0.6)	183 (0.6)	530 (0.6)		805 (1.0)	254 (1.1)	1059 (1.0)
Length of stay, mean (SD), d	4.1 (2.6)	2.9 (1.3)	3.3 (1.9)		1.9 (1.2)	3.9 (2.6)	2.3 (1.8)
Charlson Comorbidity Index score, mean (SD)	0.5 (0.9)	1.1 (1.3)	0.7 (1.1)		0.6 (1.1)	1.4 (1.7)	0.8 (1.3)
Payer							
Self	204 (0.4)	34 (0.1)	238 (0.3)		225 (0.3)	22 (0.1)	247 (0.2)
Medicare	23 544 (43.5)	23 910 (77.1)	47 454 (55.8)		40 909 (49.8)	18 480 (81.7)	59 389 (56.7)
Medicaid	2005 (3.7)	1086 (3.5)	3091 (3.6)		3966 (4.8)	984 (4.4)	4950 (4.7)
Commercial	28 075 (51.9)	5879 (19.0)	33 954 (39.9)		36 782 (44.8)	3071 (13.6)	39 853 (38.0)
Unknown	269 (0.5)	115 (0.4)	384 (0.4)		317 (0.4)	72 (0.3)	389 (0.4)
Hospital beds, No.							
<90	6374 (11.8)	2321 (7.5)	8695 (10.2)		13 342 (16.2)	1709 (7.6)	15 051 (14.4)
90 to <197	6116 (11.3)	4696 (15.1)	10 812 (12.7)		11 115 (13.5)	4640 (20.5)	15 755 (15.0)
197 to <325	17 287 (32.0)	10 892 (35.1)	28l179 (33.1)		22 647 (27.6)	6500 (28.7)	29 147 (27.8)
≥325	24 320 (45.0)	13 115 (42.3)	37 435 (44.0)		35 095 (42.7)	9780 (43.2)	44 875 (42.8)
Hospital region, No./total No. (%)^a^							
1	13 081/20 599 (63.5)	7518/20 599 (36.5)	20 599/85 121 (24.2)		18 665/23 846 (78.3)	5181/23 846 (21.7)	23 846/104 828 (22.8)
2	3785/5883 (64.3)	2098/5883 (35.7)	5883/85 121 (6.9)		5392/7271 (74.2)	1879/7271 (5.8)	7271/104 828 (6.9)
3	1999/2996 (66.7)	997/2996 (33.3)	2996/85 121 (3.5)		2911/3727 (78.1)	816/3727 (21.9)	3727/104 828 (3.6)
4	3905/5357 (72.9)	1452/5357 (27.1)	5357/85 121 (6.3)		5277/6516 (81.0)	1239/6516 (10.0)	6516/104 828 (6.2)
5	10 894/14 090 (77.3)	3196/14 090 (22.7)	14 090/85 121 (16.6)		13 937/16 655 (83.7)	2718/16 655 (16.3)	16 655/104 828 (15.9)
6	2901/4819 (60.2)	1918/4819 (39.8)	4819/85 121 (5.7)		4376/5940 (73.7)	1564/5940 (26.3)	5940/104 828 (5.7)
7	5032/8784 (57.3)	3752/8784 (42.7)	8784/85 121 (10.3)		8964/11 506 (77.9)	2542/11 506 (22.1)	11 506/104 828 (11.0)
8	7934/14 038 (56.5)	6104/14 038 (43.5)	14 038/85 121 (16.5)		15 697/19 487 (80.6)	3790/19 487 (19.5)	19 487/104 828 (18.6)
9	4566/5688 (80.3)	1150/5688 (19.7)	5688/85 121 (10.1)		10 058/11 059 (90.1)	1001/11 059 (9.9)	11 059/104 828 (9.4)
Hospital type							
Specialty acute	4538 (8.4)	1150 (3.7)	5688 (6.7)		10 058 (12.1)	1001 (4.4)	11 059 (10.4)
General acute	49 559 (91.6)	29 874 (96.3)	79 433 (93.3)		72 141 (87.8)	21 628 (95.6)	93 769 (89.5)

Abbreviation: IPAC, institutional postacute care.

^a^ Hospital region reports number, total number, and percentages to demonstrate the change in discharges to different postacute care during the 2 periods by region. A map of the regions appears in the eFigure in the Supplement.

**Table 2.** Use of Postacute Care After Lower Extremity Joint Replacement in the Preperiod and Postperiod, Stratified by Hospital Participation in Bundled Payments and Payer

Characteristic	No. (%)					
	Home			Institutional postacute care^a^		
	Home	Home health	Total	SNF	IRF	Total
Preperiod total	15 302 (18.0)	38 795 (45.6)	54 097 (63.6)	22 684 (26.7)	8280 (9.7)	31 024 (36.5)
Bundled payments^b^						
Participant	5959 (15.7)	17 347 (45.7)	23 306 (61.3)	10 885 (28.7)	372 (9.9)	14 683 (38.7)
Nonparticipant	9343 (19.8)	21 448 (45.5)	30 791 (65.3)	11 799 (25.0)	4528 (9.6)	16 341 (34.7)
Payer						
Commercial	8291 (24.4)	19 784 (58.3)	28 075 (82.7)	4603 (13.6)	1267 (3.7)	5879 (17.3)
Medicare	6221 (13.1)	17 323 (36.5)	23 544 (49.6)	17 116 (36.1)	6743 (14.2)	23 910 (50.4)
Medicaid	599 (19.4)	1406 (45.5)	2005 (64.9)	852 (27.6)	234 (7.6)	1086 (35.1)
Self	80 (33.6)	124 (52.1)	204 (85.7)	22 (9.2)	12 (5.0)	34 (14.3)
Unknown	111 (28.9)	158 (41.1)	269 (70.1)	91 (23.7)	24 (6.3)	115 (29.9)
Postperiod total	35 194 (33.6)	47 005 (44.8)	82 199 (78.4)	18 275 (17.4)	4310 (4.1)	22 629 (21.6)
Bundled payments						
Participant	12 616 (27.6)	23 445 (51.3)	36 061 (79.0)	8090 (17.7)	1492 (3.3)	9612 (21.0)
Nonparticipant	22 578 (38.2)	23 560 (39.8)	46 138 (78.0)	10 185 (17.2)	2818 (4.8)	13 017 (22.0)
Payer						
Commercial	17 266 (43.3)	19 516 (49.0)	36 782 (92.3)	2455 (6.2)	612 (1.5)	3071 (7.7)
Medicare	16 445 (27.7)	24 464 (41.2)	40 909 (68.9)	14 868 (25.0)	3573 (6.0)	18 480 (31.1)
Medicaid	1235 (24.9)	2731 (55.2)	3966 (80.1)	867 (17.5)	116 (2.3)	984 (19.9)
Self	115 (46.6)	110 (44.5)	225 (91.1)	19 (7.7)	<10	22 (8.9)
Unknown	133 (34.2)	184 (47.3)	317 (81.5)	6 (17.0)	<10	72 (18.5)

Abbreviations: IRF, inpatient rehabilitation facility; SNF, skilled nursing facility.

^a^ Long-term acute care hospitals accounted for fewer than 104 patients (<0.1%) total across the time period and are not included in the table, although they are included in the analysis.

^b^ Participant or nonparticipant status refers to any participation in bundled payments (eg, Bundled Payments for Care Improvement and/or Comprehensive Care for Joint Replacement).

After propensity matching (Table 3; eTable 1 in the Supplement), group 2 (those discharged home in the postperiod and to institutional postacute care in the preperiod) demonstrated relatively larger differences in outcome rates comparing the preperiod with the postperiod (30-day readmissions: 4980 patients [7.2%] vs 2009 patients [2.9%]; 90-day readmissions 8648 [12.6%] vs 4549 [6.6%]; complications: 584 [0.8%] vs 414 [0.6%]; 30-day mortality: 192 [0.3%] vs 58 [0.1%]; 90-day mortality: 497 [0.7%] vs 135 [0.2%]) (Table 4). In contrast, differences in unadjusted outcomes between the 2 periods were smaller in the preperiod vs postperiod for group 3 (ie, those discharged to institutional postacute care in both periods) (30-day readmissions: 2421 [10.7%] vs 2181 [9.6%]; 90-day readmissions: 4116 [18.2%] vs 3821 [16.9%]; complications: 573 [2.5%] vs 597 [2.6%]). Mortality rates did not change (30-day mortality: 402 [1.8%] vs 411 [1.8%]; 90-day mortality: 929 [4.1%] vs 935 [4.1%]).

**Table 3.** Propensity Matching for Difference-in-Differences Analysis

Characteristic	No. (%)					
	Patients shifted from IPAC to home (n = 68 891)			Patients in IPAC in both periods (n = 22 629)		
	Preperiod	Postperiod	SMD, %^a^	Preperiod	Postperiod	SMD, %^a^
Discharged to IPAC	Yes	No	NA	Yes	Yes	NA
Age, mean (SD), y	64.9 (27.0)	65.2 (9.4)	0.0	76.2 (19.1)	75.3 (11.0)	−0.1
Men	27 778 (40.3)	29 470 (42.8)	5.0	6266 (27.7)	6616 (29.2)	3.1
Member of minority racial group	5384 (7.8)	5460 (7.9)	0.4	2301 (10.2)	2725 (12.0)	7.0
Hispanic ethnicity	349 (0.5)	609 (0.9)	4.5	111 (0.5)	254 (1.1)	7.6
Charlson Comorbidity Index score						
0	44 323 (64.3)	44 242 (64.2)	−0.2	8735 (38.6)	8071 (35.7)	−6.1
1	16 379 (23.8)	16 093 (23.4)	−1.0	6876 (30.4)	6459 (28.5)	−4.3
2	5251 (7.6)	5169 (7.5)	−0.5	3717 (16.4)	3634 (16.1)	−1.4
3	1623 (2.4)	1688 (2.5)	0.6	1777 (7.9)	1951 (8.6)	5.0
4	813 (1.2)	913 (1.3)	1.3	901 (4.0)	1174 (5.2)	10.8
5	273 (0.4)	444 (0.6)	3.5	330 (1.5)	670 (3.0)	20.9
≥6	229 (0.3)	342 (0.5)	2.6	293 (1.3)	670 (3.0)	25.9
Payer						
Medicare	37 248 (54.1)	34 790 (50.5)	−7.1	19 023 (84.1)	18 480 (81.7)	−4.8
Medicaid	2182 (3.2)	2873 (4.2)	5.3	563 (2.5)	984 (4.4)	9.9
Commercial or other	29 461 (42.8)	31 228 (45.3)	5.2	3043 (13.4)	3165 (14.0)	1.1
Hospital size						
<90 beds	8641 (12.5)	10 965 (15.9)	9.7	1417 (6.3)	1709 (7.6)	3.7
91 to <197 beds	8487 (12.3)	8981 (13.0)	2.2	4238 (18.7)	4640 (20.5)	5.3
197 to <325 beds	20 481 (29.7)	18 927 (27.5)	−5.0	7294 (32.2)	6500 (28.7)	−7.8
≥325 beds	31 282 (45.4)	30 018 (43.6)	−3.7	9680 (42.8)	9780 (43.2)	0.9
Region^b^						
1	17 747 (25.8)	16 264 (23.6)	−5.0	5621 (24.8)	5181 (22.9)	−4.5
2	3637 (5.3)	4121 (6.0)	3.0	1623 (7.2)	1879 (8.3)	4.9
3	1534 (2.2)	2295 (3.3)	6.7	601 (2.7)	816 (3.6)	5.8
4	3699 (5.4)	4169 (6.1)	2.9	1077 (4.8)	1239 (5.5)	3.1
5	9945 (14.4)	11 660 (16.9)	6.9	2348 (10.4)	2718 (12.0)	4.5
6	3420 (5.0)	3545 (5.1)	0.8	1628 (7.2)	1564 (6.9)	−1.3
7	7918 (11.5)	7531 (10.9)	−1.8	2970 (13.1)	2542 (11.2)	−6.0
8	14 062 (20.4)	13 711 (19.9)	−1.3	3868 (17.1)	3790 (16.8)	−0.9
9	6929 (10.1)	5595 (8.1)	−6.7	2893 (12.8)	2900 (12.8)	0.1
Hospital type						
General acute	62 445 (90.6)	60 378 (87.6)	−9.7	21 914 (96.8)	21 628 (95.6)	−4.1
Specialty or ambulatory	6446 (9.4)	8513 (12.4)	9.7	715 (3.2)	1001 (4.4)	4.1

Abbreviations: IPAC, institutional postacute care; SMD, standardized mean difference.

^a^ SMDs are calculated as 100 × (mean_1_ – mean_0_) ÷ [(variance_1_ + variance_0_) ÷ 2]^1/2^, where 1 indicates the postperiod subgroup and 0 indicates the preperiod subgroup. The range is from −100 to 100; values between −20 and 20 are commonly used to signify satisfactory propensity matching.

^b^ A map of regions appears in the eFigure in the Supplement.

**Table 4.** Unadjusted and Adjusted Outcomes by Postacute Care Type

Outcome	No. (%)				DID estimate (95% CI)	
	Patients switched from IPAC to home		Patients in IPAC in both periods		Unadjusted	Adjusted^a^
	Preperiod	Postperiod	Preperiod	Postperiod		
Readmissions						
30-d	4980 (7.2)	2009 (2.9)	2421 (10.7)	2181 (9.6)	−3.3 (−4.6 to −1.9)^b^	−2.9 (−4.2 to −1.6)^b^
90-d	8648 (12.6)	4549 (6.6)	4116 (18.2)	3821 (16.9)	−4.6 (−6.6 to −2.7)^b^	−3.9 (−5.8 to −2.0)^b^
Complications	584 (0.8)	414 (0.6)	573 (2.5)	597 (2.6)	−0.4 (−0.9 to 0.2)	−0.3 (−0.8 to 0.3)
Mortality						
30-d	192 (0.3)	58 (0.1)	402 (1.8)	411 (1.8)	−0.2 (−0.6 to 0.1)	−0.1 (−0.4 to 0.2)
90-d	497 (0.7)	135 (0.2)	929 (4.1)	935 (4.1)	−0.6 (−1.2 to 0.1)	−0.3 (−0.9 to 0.3)

Abbreviations: DID, difference-in-differences; IPAC, institutional postacute care.

^a^ Adjustment was for any residual differences in the variables in Table 1.

^b^ *P* < .05.

In the adjusted difference-in-differences analysis, comparisons of changes between the matched groups of similar patients discharged home in the postperiod but to institutional postacute care in the preperiod (group 2) and changes of patients discharged to institutional postacute care in both periods (group 3) showed an associated decrease in readmission rates (30-day: difference, −2.9; 95% CI, −4.2 to −1.6; 90-day: difference, −3.9; 95% CI, −5.8 to −2.0). However, there were no differential changes in complication (difference, −0.3, 95% CI, −0.8 to 0.3) or mortality rates (30-day: difference, −0.2; 95% CI, −0.5 to 0.2; 90-day: difference, −0.4; 95% CI, −1.0 to 0.2).

In a sensitivity analysis restricted to Medicare patients only, similar significant decreases in readmission rates at 30 and 90 days were noted (eTable 2 in the Supplement). There were no differences in estimates for any outcome comparing hospitals that did and did not participate in BPCI and CJR in sensitivity analyses (eTable 3 in the Supplement). The overall estimates for changes in outcomes did vary by age and comorbidity in adjusted difference-in-differences analyses: there were no significant differences in estimates for any outcome in the youngest patients. Patients with the lowest comorbidity levels were associated with significantly larger decreases in readmission rates but no changes in other outcomes. In contrast, patients in the oldest age group and with the highest comorbidity level were associated with significant differential changes in readmission rates and mortality but also significant increases in complication rates (eTable 4 in the Supplement). The E-value analysis suggested that an unmeasured confounder was unlikely to easily explain the readmission or complication results, but mortality results were more vulnerable to confounding (eTable 5 in the Supplement).

## Discussion

In this study examining secular changes in discharge patterns due to payment reforms among patients undergoing LEJR surgery, switching patients from a discharge to institutional postacute care to a discharge home did not seem to be associated with adverse outcomes. Conversely, in contrast to our hypothesis, we found significant associated reductions in 30-day and 90-day readmissions. Our results suggest that adults discharged home in 2016 to 2018 who were clinically similar to adults discharged to institutional forms of postacute care (most commonly SNF) in 2011 to 2013 were not more likely to sustain potentially adverse outcomes, such as hospital readmissions, surgical complications, or mortality. These findings were similar across payers and hospitals, whether participating in bundled payments (as a particularly relevant type of payment reform) or not.

These findings are important for clinicians and policy makers seeking to deliver high-value postoperative care for patients undergoing LEJR and are important for patients themselves. Since value-based payment models such as bundled payments and accountable care organizations incentivize more home care after surgery, a major concern is that financial accountability will lead to reductions in care delivery that could lead to unintended adverse effects. This is particularly concerning for patients who are switched from a higher level of postacute care to being discharged home with less support. Thus, our findings are an important data point that policy reforms intended to reduce intensity of postacute care for LEJR may not be harming patients. Further, our results are consistent with model-specific studies suggesting that neither bundled payments nor HRRP for LEJR were associated with harm in frail populations, despite significant changes in postacute care use.^6,8,12,13^ In addition to examining the issue more broadly across all payers and patients receiving LEJR within a state, our study examined the most relevant patient population for whom unintended effects from switching the level of postacute care may occur: those who used to be discharged to institutional postacute care and are now discharged home.

### Limitations and Strengths

This study has limitations. It is important to keep the context of this observational study in mind when interpreting these findings. For example, our results provide limited insight into underlying mechanisms. While an interpretation is that these patients did not require institutional postacute care to begin with, changes in surgical care (such as more intensive pre-operative and postoperative physical therapy or more intensive medical management) that are not observable in our data could explain reductions in readmission rates in the postperiod. Similarly, although we matched patients on factors that accurately estimated the likelihood of a patient being discharged to a particular postacute care setting, selection of healthier patients for surgery in the postperiod in ways that are not observed in our data could also play a role in our results. For example, we could not measure frailty or social supports directly. Changes in patient selection may play a large role in cost savings in bundled payment programs, for example.^10^ Quality improvement or other initiatives may also have played a role in changing both postacute care use and outcomes. Given concerns regarding unmeasured confounding, we caution against interpreting findings as demonstrating reduced harm; rather, we interpret these findings as not showing an association with increased harm when institutional postacute care use is reduced. We did find higher susceptibility to unmeasured confounding particularly in our sensitivity analyses (ie, relatively low E-values, indicating that small magnitudes of unmeasured confounding could change the results, if present). While our data set captures surgical procedures, hospitalizations, complications, and mortality, it does not capture ambulatory care such as emergency department visits or observation stays. While our patient population has similar demographic characteristics as patients undergoing LEJR procedures nationally, our data are derived from 1 state, and results may vary in other states depending on their policies or utilization patterns.

In addition, our results may not apply to other postacute care populations. Another study analyzing a general population of Medicare beneficiaries (including both medical and surgical patients) found patients discharged to SNF had lower 30-day readmission rates than patients receiving home health.^17^ One reason our findings may differ is that our study population (patients who underwent LEJR) may have substantially different needs for postacute care supports than medical populations. Future work is needed to delineate the best postacute care setting for individual patients based on their characteristics and in-hospital treatment.

Strengths of our study include the use of a representative sample of all elective total joint arthroplasties across payers, surgical sites (including ambulatory surgical centers and specialty surgical hospitals), and both participating and nonparticipating hospitals. Our statistical approach combined the strengths of propensity matching with a difference-in-differences analysis to isolate similar cohorts of patients in the preperiod and postperiod and compare outcomes, adjusting for patient and hospital factors as well as temporal factors that could confound results. However, in addition to the limitations noted above, we were also unable to evaluate the intensity or quality of postacute care delivered at home or in institutional postacute care settings. Although Pennsylvania is a diverse state, findings may vary in other states with different penetration of bundled payments, market competition, and population of adults undergoing surgery.

## Conclusions

Reductions in postacute care use and the use of less expensive forms of postacute care are a common source of savings in value-based payment models, leading some to refer to postacute care as the piggy bank for savings.^5^ In this context, it is increasingly important to monitor for potential unintended consequences in patients leaving the hospital and to ensure policies maximize value and minimize harm. During a time period in which changes in financial incentives likely spurred observed changes in postacute care following LEJR surgery, the findings of this study suggest that increases in discharges home did not seem to be associated with increased harm.

## Notes

Supplement. **eFigure.** Regional Map of PHC4 Hospitals **eTable 1.** Propensity to Be Discharged Home in Preperiod and Postperiod **eTable 2.** Unadjusted and Adjusted Outcomes by Postacute Care Type, Restricted to Medicare Cohort **eTable 3.** Outcomes at BPCI- and CJR-Participating and Nonparticipating Hospitals **eTable 4.** Outcomes for Low and High Age and Comorbidity Quartiles **eTable 5.** E-value Analysis for Potential Unmeasured Confounders
