# Supplementary material for: Association of Discharge to Home vs Institutional Postacute Care With Outcomes After Lower Extremity Joint Replacement
Source: JAMA Netw Open. 2020 Oct 23;3(10):e2022382. doi: 10.1001/jamanetworkopen.2020.22382 (PMC7584947; doi:10.1001/jamanetworkopen.2020.22382)
Supplement: Supplement. — eFigure. Regional Map of PHC4 Hospitals eTable 1. Propensity to Be Discharged Home in Preperiod and Postperiod eTable 2. Unadjusted and Adjusted Outcomes by Postacute Care Type, Restricted to Medicare Cohort eTable 3. Outcomes at BPCI- and CJR-Participating and Nonparticipating Hospitals eTable 4. Outcomes for Low and High Age and Comorbidity Quartiles eTable 5. E-value Analysis for Potential Unmeasured Confounders [file jamanetwopen-e2022382-s001.pdf]

Supplemental Online Content

Burke RE, Canamucio A, Medvedeva E, Hume EL, Navathe AS. Association of discharge to home vs institutional postacute care with outcomes after lower extremity joint replacement. *JAMA Netw Open*. 2020;3(10):e2022382. doi:10.1001/jamanetworkopen.2020.22382

eFigure. Regional Map of PHC4 Hospitals
eTable 1. Propensity to Be Discharged Home in Preperiod and Postperiod
eTable 2. Unadjusted and Adjusted Outcomes by Postacute Care Type, Restricted to Medicare Cohort
eTable 3. Outcomes at BPCI- and CJR-Participating and Nonparticipating Hospitals
eTable 4. Outcomes for Low and High Age and Comorbidity Quartiles
eTable 5. E-value Analysis for Potential Unmeasured Confounders

This supplemental material has been provided by the authors to give readers additional information about their work.

© 2020 Burke RE et al. *JAMA Network Open.*

eFigure. Regional Map of PHC4 Hospitals

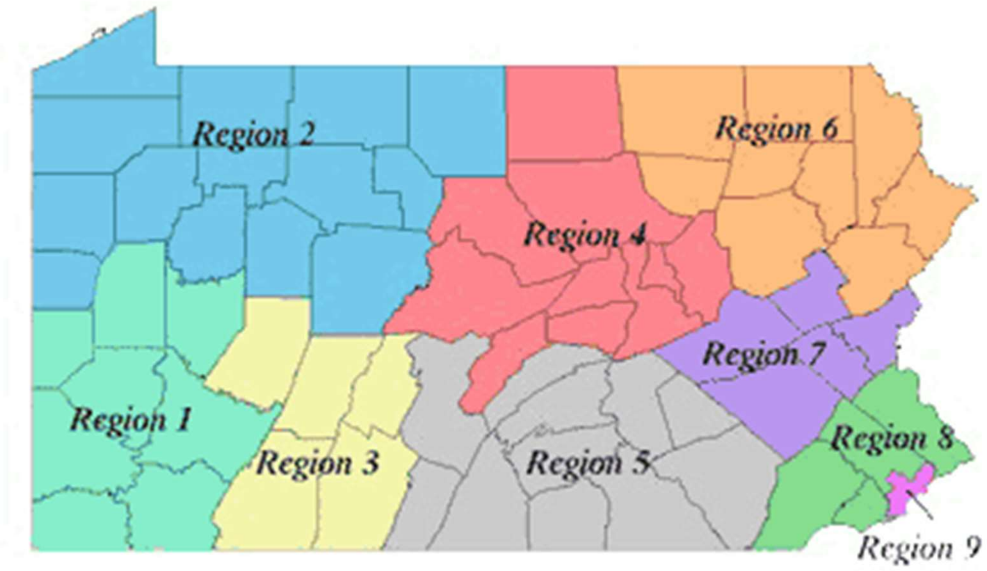

Map can be located at: http://www.phc4.org/dept/dc/state.htm.

© 2020 Burke RE et al. *JAMA Network Open.*

eTable 1. Propensity to Be Discharged Home in Preperiod and Postperiod

	Pre-period	Post-period
	Odds ratio	Odds ratio
Age	0.92	0.91
Male	1.89	1.71
Race		
White	-	-
Non-white	0.61	0.58
Hispanic	0.97	0.82
Charlson Index		
0	-	-
1	0.64	0.53
2	0.47	0.37
3	0.33	0.25
4	0.25	0.24
5	0.17	0.20
6 or more	0.18	0.12
Primary Payer		
Self, Commercial, unknown	-	-
Medicare	0.68	0.75
Medicaid	0.33	0.32
Bed Count		
<90	0.74	1.00
90<197	0.63	0.54
197<325	0.86	0.91
>325	-	-
Region		
1	1.93	1.70
2	2.37	1.22
3	2.59	1.57
4	3.53	2.16
5	4.40	2.54
6	1.90	1.46
7	1.26	1.36
8	1.40	2.08
9	-	-
Facility type		
General Acute	0.40	0.65
Specialty acute or ambulatory surgery	-	-
**C-statistic**	**0.81**	**0.82**

Models to calculate the propensity score for the pre-period cohort applied to the post-period cohort (to create Group 1, left hand column), and to calculate the propensity score in the post-period applied to the pre-period (to create Groups 2 and 3, right-hand column) are displayed.

© 2020 Burke RE et al. *JAMA Network Open.*

eTable 2. Unadjusted and Adjusted Outcomes by Postacute Care Type, Restricted to Medicare Cohort

Outcomes	iPAC pre-period, home post-period (Group 2)		iPAC both time periods (Group 3)		Unadjusted DID (95% CI)	Adjusted DID (95% CI)
	Pre	Post	Pre	Post		
30-day readmission	7.5	3.7	11.3	10.1	**-2.59 (-4.29, -0.89)**	**-2.40 (-4.04, -0.75)**
90-day readmission	13.4	7.4	19.1	17.5	**-4.46 (-6.61, -2.32)**	**-3.92 (-5.99, -1.85)**
30-day mortality	0.4	0.1	2.0	2.1	-0.40 (-0.84, 0.04)	-0.40 (-0.82, 0.02)
90-day mortality	1.1	0.3	4.6	4.6	**-0.82 (-1.52, -0.13)**	**-0.83 (-1.48, -0.17)**

I-PAC = institutional post-acute care. DID = Difference in differences estimate, CI = confidence interval. **Bold** connotes statistical significance. The analysis was adjusted for age, sex, race, ethnicity, Charlson score, payer, beds, and type of hospital and for clustering at the hospital level. While this analysis also demonstrated a slightly lower 90-day mortality rate, our E-value analysis suggests these results should be viewed as potentially confounded by unmeasured confounders.

© 2020 Burke RE et al. *JAMA Network Open.*

eTable 3. Outcomes at BPCI- and CJR-Participating and Nonparticipating Hospitals

Outcomes	Participating		Non-participating		Unadjusted DID (95% CI)	Adjusted DID (95% CI)
	Pre	Post	Pre	Post		
30-day readmission	6.0	4.8	5.9	4.2	0.55 (-0.13, 1.23)	0.67 (-0.03, 1.38)
90-day readmission	11.1	9.3	10.9	8.6	0.45 (-0.49, 1.37)	0.62 (-0.30, 1.54)
30-day mortality	0.5	0.5	0.5	0.5	0.0 (-0.15, 0.14)	0.02 (-0.12, 0.15)
90-day mortality	1.2	1.1	1.3	1.0	0.19 (-0.05, 0.42)	0.24 (-0.00, 0.49)

I-PAC = institutional post-acute care. DID = Difference in differences estimate, CI = confidence interval. **Bold** connotes statistical significance. The analysis was adjusted for age, sex, race, ethnicity, Charlson score, payer, beds, and type of hospital and for clustering at the hospital level.

© 2020 Burke RE et al. *JAMA Network Open.*

eTable 4. Outcomes for Low and High Age and Comorbidity Quartiles

Outcomes	I-PAC pre-period, home post-period (Group 2)		I-PAC both time periods (Group 3)		Adjusted DID (95% CI)
	Pre	Post	Pre	Post	
**30-day readmission**					
Younger age	6.67	2.37	8.81	7.29	-1.81 (-4.26, 0.64)
Older age	10.46	5.27	12.31	11.32	**-3.89 (-6.41, -1.36)**
Low comorbidity	6.20	2.21	7.81	6.46	**-2.69 (-4.40, -0.98)**
High comorbidity	12.38	5.49	1.32	13.87	**-5.45 (-8.17, -2.74)**
**90-day readmission**					
Younger age	12.97	6.38	15.43	14.12	-3.16 (-6.84, 0.53)
Older age	17.49	9.03	20.88	19.42	**-6.47 (-10.10, -2.85)**
Low comorbidity	10.59	5.57	12.22	10.97	**-3.76 (-6.37, -1.15)**
High comorbidity	22.46	10.61	26.82	23.82	**-8.49 (-11.80, -5.17)**
**Complication**					
Younger age	0.97	1.61	2.28	2.35	0.68 (-0.77, 2.13)
Older age	3.88	2.03	7.44	2.73	**3.12 (1.89, 4.35)**
Low comorbidity	1.36	1.69	2.18	1.92	0.54 (-0.45, 1.54)
High comorbidity	5.50	1.71	9.90	2.95	**2.88 (1.19, 4.58)**
**30-day mortality**					
Younger age	0.03	0.06	0.35	0.27	0.15 (-0.51, 0.82)
Older age	1.16	0.29	2.96	3.14	**-0.83 (-1.60, -0.06)**
Low comorbidity	0.09	0.04	0.87	0.55	0.25 (-0.27, 0.76)
High comorbidity	1.32	0.28	3.56	3.41	**-1.20 (-2.16,-0.24)**
**90-day mortality**					
Younger age	0.19	0.14	0.57	0.76	-0.14 (-0.90,0.63)
Older age	2.88	0.59	6.97	7.06	**-1.85 (-2.98, -0.71)**
Low comorbidity	0.26	0.09	1.57	1.15	0.19 (-0.47, 0.84)
High comorbidity	3.04	0.68	8.31	7.73	**-2.35 (-3.82, -0.88)**

Age and Charlson comorbidity score were divided into quartiles in our cohort and their outcomes compared. The lowest age quartile included ages 18-61, the oldest included ages 77-100. The lowest Charlson quartile included a score of zero, the highest included scores of 2-15. Bold indicates statistical significance. In the case of complications, although the DID is positive, the complication rates decreased in both settings; they decreased more in Group 3 (those discharged to institutional post-acute care in both time periods) than in Group 2, hence the positive score.

© 2020 Burke RE et al. *JAMA Network Open.*

eTable 5. E-value Analysis for Potential Unmeasured Confounders

Outcomes	E-value
30-day readmission	150.2
90-day readmission	101.7
Complication	15.6
30-day mortality	6.2
90-day mortality	9.4

The E-value represents the minimum strength that an unmeasured confounder would need to have with both treatment and outcome to fully explain away a specific treatment-outcome association, conditional on the measured covariates. Although its use in difference-in-differences analyses has not been well-described, we used the "difference in continuous outcomes" effect measure, calculated as E-value = RR* + sqrt[RR* *x* (RR* − 1)] where sqrt= square root. RR* is calculated as 1/(exp(0.91*d)), where d= standardized effect size (mean of the outcome divided by the standard deviation of the outcome). The results for 30-day readmission, for example, suggest that our findings could be explained away by an unmeasured confounder associated with both the treatment and the outcome by 150-fold, while for our mortality estimates, such a confounder would only require associations with treatment and outcome by 6.2-fold.

© 2020 Burke RE et al. *JAMA Network Open.*
